# Analysis of mutational dynamics at the *DMPK* (CTG)_n_ locus identifies saliva as a suitable DNA sample source for genetic analysis in myotonic dystrophy type 1

**DOI:** 10.1371/journal.pone.0216407

**Published:** 2019-05-02

**Authors:** Eyleen Corrales, Melissa Vásquez, Baili Zhang, Carolina Santamaría-Ulloa, Patricia Cuenca, Ralf Krahe, Darren G. Monckton, Fernando Morales

**Affiliations:** 1 Instituto de Investigaciones en Salud (INISA), Universidad de Costa Rica, San José, Costa Rica; 2 Centro de Investigación en Neurociencias, Universidad de Costa Rica, San José, Costa Rica; 3 Department of Genetics, University of Texas MD Anderson Cancer Center, Houston, Texas, United States of America; 4 Institute of Molecular, Cell and Systems Biology, College of Medical, Veterinary and Life Sciences, University of Glasgow, Glasgow, United Kingdom; Sanford Burnham Prebys Medical Discovery Institute, UNITED STATES

## Abstract

Genotype-to-phenotype correlation studies in myotonic dystrophy type 1 (DM1) have been confounded by the age-dependent, tissue-specific and expansion-biased features of somatic mosaicism of the expanded CTG repeat. Previously, we showed that by controlling for the confounding effects of somatic instability to estimate the progenitor allele CTG length in blood DNA, age at onset correlations could be significantly improved. To determine the suitability of saliva DNA as a source for genotyping, we used small pool-PCR to perform a detailed quantitative study of the somatic mutational dynamics of the CTG repeat in saliva and blood DNA from 40 DM1 patients. Notably, the modal allele length in saliva was only moderately higher in saliva and not as large as previously observed in most other tissues. The lower boundary of the allele distribution was also slightly higher in saliva than it was in blood DNA. However, the progenitor allele length estimated in blood explained more of the variation in age at onset than that estimated from saliva. Interestingly, although the modal allele length was slightly higher in saliva, the overall degree of somatic variation was typically lower than in blood DNA, revealing new insights into the tissue-specific dynamics of somatic mosaicism. These data indicate that saliva constitutes an accessible, non-invasive and suitable DNA sample source for performing genetic studies in DM1.

## Introduction

Myotonic dystrophy type 1 (DM1) is the most common dominantly inherited myopathy in adults. It is a progressive and disabling disease that shows a highly variable phenotype, both in severity and clinical manifestations. The main symptoms include myotonia, muscle wasting and weakness, cardiac problems, cataracts, somnolence, cognitive dysfunction and behavioral abnormalities [1, 2]. The disease is caused by the expansion of an unstable trinucleotide (CTG)_n_ repeat, located in the 3'-untranslated region (3'-UTR) of the DM protein kinase (*DMPK*) gene [3–5]. Non-DM1 individuals in the general population usually carry between 5 to 37 CTG repeats, while individuals with DM1 inherit from 50 to several thousand CTG repeats [3]. Above 50 CTGs the repeat becomes highly unstable, both in the germ line and somatic tissues [6, 7].

Somatic mosaicism of the expanded CTG repeat first became evident on autoradiographs obtained from Southern blot hybridization of restriction-digested genomic blood DNA. Smears instead of discrete bands were observed for the expanded alleles in DM1 patients, representing a collection of cells in the same tissue containing different repeat lengths [3, 4, 8]. For this reason, it was common practice to measure the midpoint of the smear and use this allele size in clinical correlations (genotype to phenotype). Although this allele size correlates positively with the severity of the disease and negatively with the age of onset of symptoms, these correlations typically remained poor, explaining less than 50% of the variation in age of onset [9–12].

Failure to reveal accurate clinical correlations in DM1 could be explained by omission to take into account some particular features of the somatic instability (SI), such as the tissue-, age- and allele length-dependence [13–17]; features that very likely contribute to the age of onset and the progressive nature of the disease. In order to control for some of these confounding effects of SI and to improve the clinical correlations in DM1, we previously used small pool-PCR (SP-PCR) to estimate the progenitor allele length (PAL *i*.*e*. the allele size transmitted by the affected parent to the affected offspring) in blood DNA. Results from these studies clearly indicate that the estimated PAL (ePAL) is the major modifier of the age of onset of the DM1, explaining more than 70% of the variation in age of onset. SP-PCR was also used to measure the degree of SI, showing that the residual variation in SI, not accounted for by PAL and age, also contributes towards disease progression [16, 18].

The study of multiple tissues from the same DM1 patients has revealed the presence of different modal repeat sizes between tissues. Notably, much larger expanded alleles are observed in skeletal muscle (the main affected tissue in DM1) than in the blood of DM1 patients [19–21]. It was initially thought that age at onset correlations would improve by using the allele size measured in skeletal muscle. Surprisingly though, age at onset correlations in skeletal muscle DNA were poorer than those obtained with blood DNA [21]. These data suggest that the confounding effects of somatic mosaicism are greater in muscle than they are in blood.

Here, by using SP-PCR and by comparing with peripheral blood lymphocytes (PBL), we explore the suitability of using saliva to perform the molecular diagnosis and establish age at onset correlations in DM1 through a less invasive method. Several advantages of using saliva over blood as the source of DNA for genetic studies have been described, including the ease and speed of collection, lower cost, non-invasive nature with no counter-indications [22], ease of storage and shipment, and lack of clotting [23, 24]. Because saliva collection does not involve the use of needles, it is overall more comfortable for patients [25] and more patients are willing to participate in research [26]. It is worth noting that DNA and RNA extracted from saliva has been used for many large studies, including analyses of cancer, metabolic disease, infectious disease, sports medicine, drug abuse, orthodontics, and even proteomic, transcriptomic and metabolomic studies (reviewed in [25]).

## Material and methods

### Study population

Peripheral blood and saliva samples were collected simultaneously from 40 Costa Rican DM1 patients (21 women and 19 men): three late-onset cases, 31 classic adult-onset cases, three juvenile-onset cases, two congenital-onset cases and one carrier subject who was asymptomatic at sampling. The DM1 population has already been well characterized and the age of onset has been previously recorded and reported [16, 18]. Age of onset was based on the detection of physical myotonia (grip myotonia), muscle weakness and/or the presence of cataracts. Age of onset was recorded after clinical evaluation by one of four different experienced neurologists, or after an interview by the same neurologists or by one of two different experienced clinical geneticists.

For saliva collection, in order to increase the fraction of buccal epithelium cells recovered, the patients were requested to carefully wipe the inner side of their cheeks with their tongue and spit in a collection tube until obtaining ~5 ml of saliva. Simultaneously, 10 ml of peripheral blood was drawn into EDTA-containing vacutainer tubes. DNA was isolated by proteinase K/phenol-chloroform extraction and quantified by optical density at 260 nm in a NanoDrop spectrophotometer (Thermo Scientific, USA) and stored at -20°C. The Scientific-Ethics Committee of the Universidad de Costa Rica approved the project. All samples were collected after obtaining written informed consent in accordance with the protocols approved by the Scientific-Ethics Committee of the Universidad de Costa Rica.

### Molecular analysis

#### Measuring ePAL and degree of somatic instability

To estimate the PAL and determine the degree of SI in each sample, we used SP-PCR as previously described [15, 16]. Briefly, for estimating PAL, we performed five reactions per sample with ~200 to 300 pg of input DNA and the PCR products were hybridized with a (CTG)_66_ radiolabeled probe. The PAL was estimated as the approximate lower boundary of the total allele distribution obtained for each sample [16, 18].

In order to carry out a detailed quantitative analysis of somatic mosaicism, we used single molecule SP-PCR (using 10 to 70 pg of input DNA per reaction) to measure at least 50 single molecules per sample per patient. The degree of SI was defined as the difference between the 10^th^ and 90^th^ percentile of the total allele distribution as described previously [16, 18]. SP-PCR products were detected by radioactive Southern blot hybridization and sized using UVIbandmap software (UVITEC, UK).

#### Screening for variant repeats

Previously described methods [27] were followed in order to identify the presence or absence of AciI sensitive variant repeats in the Costa Rican DM1 samples. Briefly, we carried out two PCRs per sample using 400 to 500 pg of input DNA followed by an AciI restriction digestion according to instructions provided by the manufacturer (New England Biolabs, USA). Through this approach, we were be able to exclude the most commonly observed CGG and CCG variant repeats within the CTG repeat expansion, but this does not exclude the presence of other variant repeats type in the samples analysed in this study. Digested and undigested PCR products were resolved by agarose gel electrophoresis and detected by Southern blot hybridization. A positive variant repeat sample was analysed in each experiment to confirm the presence or absence of variant repeats in the samples under investigation. The structure of the positive variant repeat allele is ~(CTG)_225_(CCG)_1_(CTG)_1_(CCG)_1_(CTG)_4_(CCG)_1_(CTG)_1_(CCG)_1_(CTG)_1_(CCG)_2_(CTG)_1_(CCG)_1_(CTG)_1_(CCG)_1_(CTG)_23_.

#### DNA methylation analyses of CTCF binding sites

Analysis of DNA methylation levels in two CTCF binding sites flanking the (CTG)_n_ repeat at the *DMPK* locus was carried out through PyroMethA technique (Pyrosequencing-based Methylation Analysis or PMA). The assays employed were designed to interrogate 11 CpG sites upstream of the CTG repeat (six within the first CTCF-binding site, ‘CTCF1’), and six CpG sites downstream of the CTG repeat (three within the second CTCF-binding site, ‘CTCF2’) [28, 29]. Firstly, 300 ng of DNA from each sample was subjected to sodium-bisulfite treatment using the EZ DNA Methylation-Gold kit (Zymo Research, USA), according to instructions provided by the manufacturer. This treatment converts unmethylated cytosines to uracils, while leaving 5-methylcytosines (5-mC) unaffected. The presence of cytosine residues (as indicative of methylation) flanking the CTG repeat expansion was later detected quantitatively through pyrosequencing. Oligonucleotides required for this purpose were custom designed using the PyroMarkQ Assay Design software 1.0 (Biotage, USA) and optimized accordingly (see S1 Table for complete list of primers used in this study).

Briefly, PCR amplification of 15 ng of bisulfite treated-DNA was carried out in a final reaction volume of 25 μl, containing 1X Hot StarTaq Master Mix (Qiagen, Germany), 100 pmol of gene-specific forward primer (either PS-DMPK-F3 for CTCF1, or PS-DMPK-F4 for CTCF2), 10 pmol of gene-specific reverse primer (either PS-U2-DMPK-R3 for CTCF1, or PS-U2-DMPK-R4 for CTCF2) and 90 pmol of biotinylated universal primer (PS-Bio-UNIV2). Amplification was performed with a denaturing step of 5 min at 95°C, followed by 45 cycles of denaturing for 30 s at 95°C, annealing for 1 min at 51°C for CTCF1 or 50°C for CTCF2, and extension for 45 s at 72°C. A final extension step was performed at 72°C for 7 min.

Amplified PCR products (8 μl) were combined with 2 μl streptavidin sepharose high-performance beads (GE Healthcare, UK), 40 μl of binding buffer (Biotage, USA) and 30 μl of MilliQ water, and subjected to single-strand isolation of the biotinylated template using the PyroMark Vacuum Prep WorkStation (Biotage, USA) as instructed by the manufacturer. Isolated products were dispensed into optical plates containing 12 μl of the corresponding sequencing primer (either PS-DMPK-S3 for CTCF1, or PS-DMPK-S4 for CTCF2) dissolved in annealing buffer (Biotage, USA) to a final concentration of 0.4 μM. To allow annealing of the sequencing primer to the template, plates were incubated for 5 min in a heating block at 85°C, left to cool for 5 min and then placed at room temperature for 5 min.

Pyrosequencing was carried out using the PSQ96 HS platform (Biotage, USA) and PyroMark Gold Q96 reagents (Biotage, USA) according to the manufacturer’s instructions and analysed with Q-CpG software (Biotage, USA), which estimates the methylation percentage for each of the interrogated CpG sites. The average methylation value of for all CpG sites analysed in each assay was calculated and the CTCF-binding sites were considered to be methylated when this value was higher than 10% [30].

### Statistical analysis

Paired sample *t-*tests were carried out in SPSS Statistics 19 (IBM, USA) in order to compare ePAL and SI among the two different sample sources, whereas single and multiple linear regressions were used to identify the major modifiers of the age of onset and the degree of SI of each tissue. Frequency curves from total allele distributions were compared through Anderson-Darling (AD) testing, using the kSamples 1.2–4 package for R.

## Results

### ePAL measured from saliva samples can be used for clinical correlations in DM1

**Fig 1:**
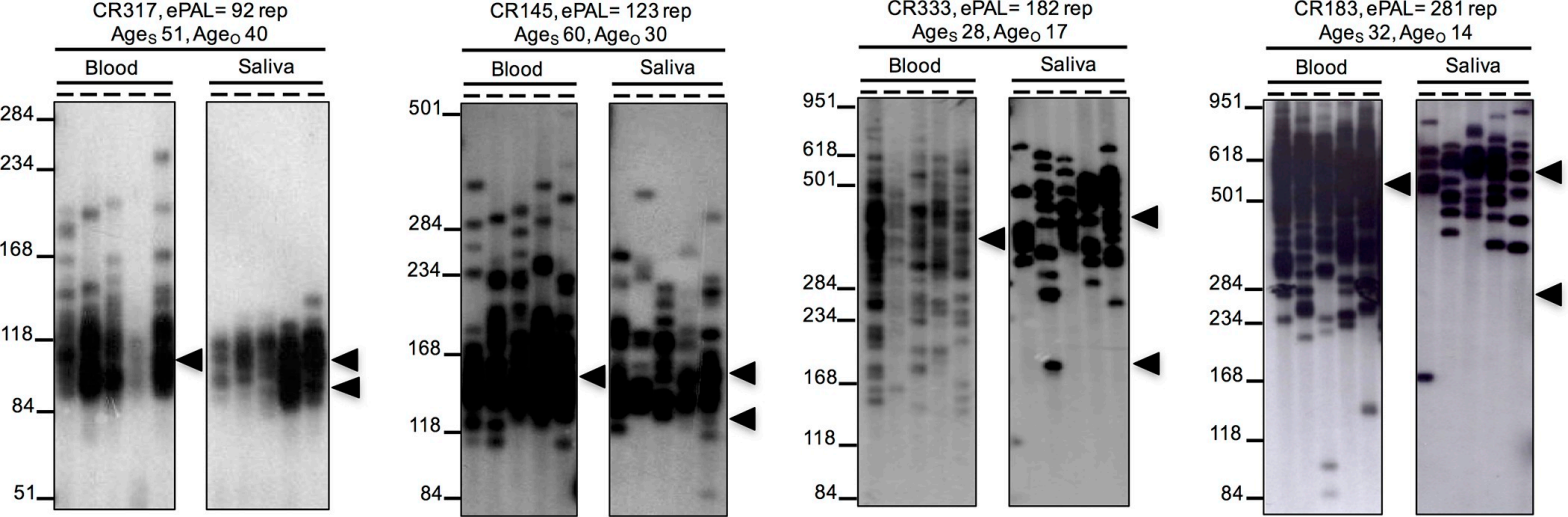
Representative autoradiographs of SP-PCR for progenitor allele estimation (ePAL) in four DM1 patients using two different DNA sources (left blood and right saliva). The lower boundary of the allele distribution in each tissue was used to estimate the PAL. The bottom arrowhead indicates the PAL estimated in blood. In patients with blood ePAL < 150 CTG repeats (CR317 and CR145), the estimation of PAL using saliva was about the same, but in patients with an ePAL > 150 CTG repeats (CR333 and CR183) the ePAL measured in saliva was larger than in blood. The top arrowhead indicates the modal allele length for each tissue. For each sample, we indicate the ePAL measured in blood (ePAL), the age at sampling (Age_s_) and the age of onset (Age_o_). The molecular weight marker sizes are shown converted to CTG repeat numbers.

**Fig 2:**
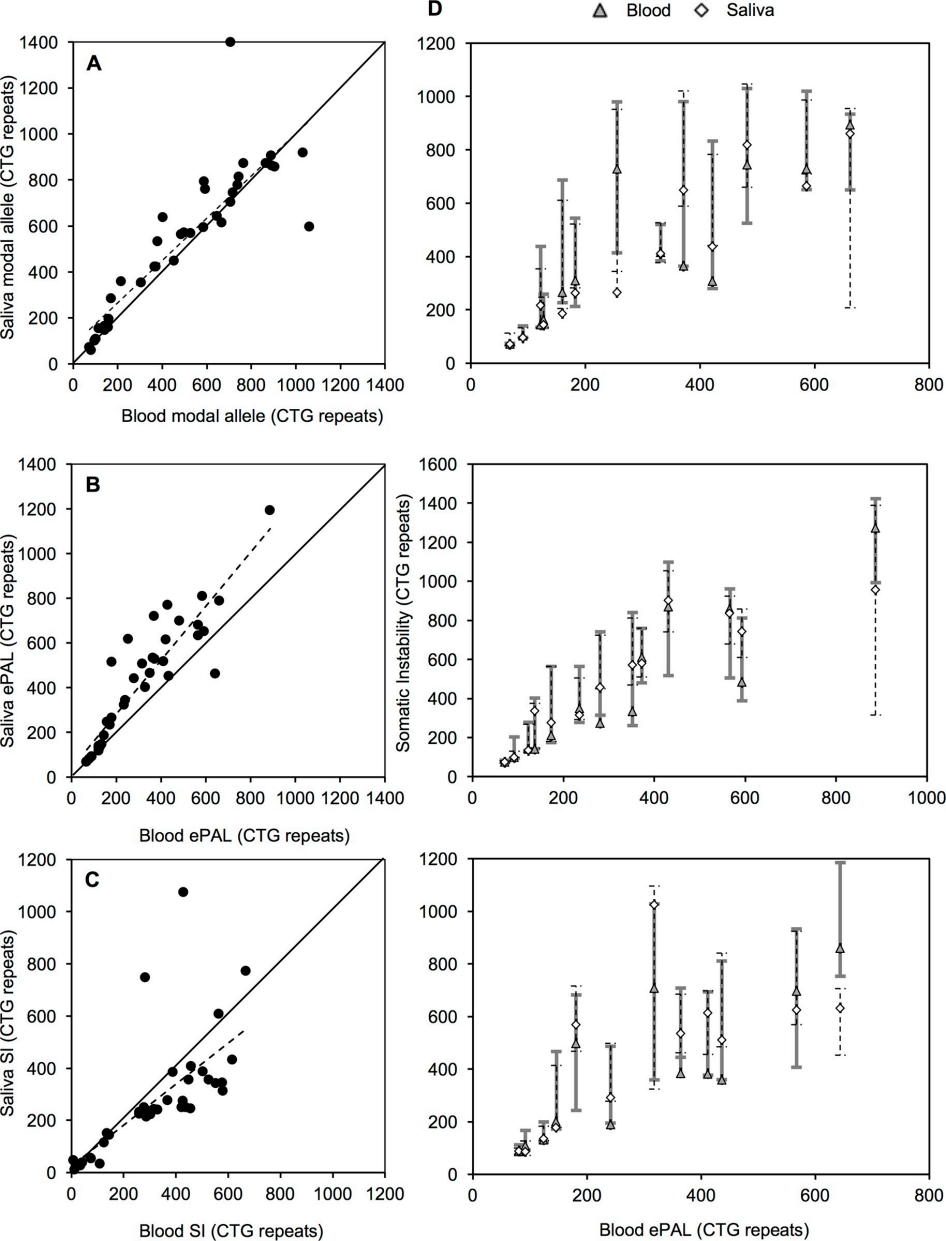
Comparison of the modal allele size, progenitor allele length (ePAL), and degree of somatic instability (SI) from two different DNA tissue sources of the same DM1 patient. Panel A shows the comparison of the modal allele length in the two tissues analysed. The dashed line corresponds to the line of best for the correlation. Points above the solid line indicate larger modal allele length in saliva than in blood. Panel B shows the comparison of the ePAL in the two tissues analysed. The dashed line corresponds to the line of best fit for the correlation. Points above the solid line indicate larger ePALs in saliva than in blood. Panel C shows the comparison of the degree of SI in the two tissues analysed. The dashed line corresponds to the line of best fit for the correlation. Points below the solid line indicate a lower degree of SI in saliva than in blood. Panels in D show a diagrammatic comparison of the somatic instability degree (SI) and the estimated progenitor allele length (ePAL) from two different DNA sources of the 38 DM1 patients analysed in this project. The whiskers represent the SI range for each tissue of each patient, whereas the diamonds and triangles indicate the modal allele in saliva and blood respectively. For better comparison, samples were split over three graphs according to the ePAL measured in blood.

By using SP-PCR we were able to amplify in all of the DM1 samples the expanded CTG allele in both blood and saliva DNA (Fig 1). We observed that the modal allele length measured in both tissues was highly correlated (*r* = 0.879, *n* = 38, *p* < 0.001, Fig 2A) and that in saliva, it was typically a little bit larger than in blood (mean modal allele in blood = 486 repeats; saliva = 529 repeats; *t* = -1.74, df = 37, *p* = 0.090, Figs 1, 2A and 2D and data in S1A Fig).

Interestingly, we identified two individuals who presented with a small non-disease associated allele (< 50 repeats) and two additional clear expanded alleles in the two tissue sources analysed (≥ 50 repeats). These patients showed the typical adult-onset form of the disease. The presence of two expanded alleles is assumed to reflect an early embryonic mutation event [31, 32], and because of the difficulty in defining the ePAL or assigning somatic variants to the appropriate allele in such individuals, these two cases were excluded from further analysis.

Previously, we estimated the PAL as the lower boundary of the allele distribution after performing SP-PCR with 200 to 300 pg of input DNA obtained from peripheral blood [16, 18]. Here, by using the same approach, we investigated if the lower boundary observed in PBL DNA was conserved in DNA derived from saliva collected at the same point in time. The PAL was estimated from both tissue sources in 40 DM1 patients (80 samples in total). We observed that blood and saliva ePALs were highly correlated (*r* = 0.908, *n* = 38, *p* < 0.001, Fig 2B). In general, the ePAL was larger in saliva than in blood (mean ePAL in blood = 310 repeats; saliva = 414 repeats; *t* = -5.32, df = 37, *p* < 0.001, Figs 1, 2B and 2D and data in S1B Fig, data in S2 Table). This difference was most evident in patients with ePALs larger than 150 CTGs for whom only one patient showed a larger ePAL from blood than saliva DNA. When the ePAL was smaller than 150 CTG repeats, the lower boundaries of the distribution of expanded alleles, and therefore the ePALs, in DNA from the two tissue sources were very closely conserved.

With the aim of determining which sample source might be more suitable for establishing genotype to phenotype correlations in DM1, we explored the relationship between ePAL and age at onset of symptoms. One mutation carrier was excluded from these analyses, as he remained asymptomatic at the time of sampling. Linear regression models showed that the logarithm of PAL estimated in blood DNA explained 75% of the variation in age at onset, whereas the logarithm of PAL estimated in saliva DNA accounted for only 66% of the variation in age of onset (Model 1, Table 1). This analysis did not reveal a significant difference (Fisher r to z transformation, *z* = -0.73, *p* = 0.465) in the coefficients of determination between blood (*r*^2^ = 0.748, *n* = 37) and saliva (*r*^2^ = 0.661, *n* = 37). A previous study has suggested the presence of additional nonlinear components in the regression models of age of onset and the size of the ePAL [16]. Thus, we included a quadratic component into the model, but this did not lead to any significant improvement (Model 2, Table 1). Given that the modal allele length in saliva DNA was greater than that observed in blood, it suggests that the net average rate of expansion is greater in saliva than in blood. Likewise, the larger PAL estimated from saliva also suggests that the lower boundary has increased more rapidly in this tissue. This interpretation is consistent with the greater explanatory power of blood ePAL in defining genotype to phenotype correlations and suggests the PAL estimated from blood is likely to be closer to the true PAL than that estimated from saliva.

**Table 1:** Regression models of the relationship between age at onset (Age_o_) and the progenitor allele length (ePAL) estimated from two different DNA tissue sources of the same DM1 patient.

Model	Source	Adjusted *r*^2^	*p*	Parameter		Coefficient	Standard error	*t*-statistic	*p*
Model 1: Age_o_ = β_0 _+ β_1_ log(ePAL) *n* = 37 individuals	Blood	0.748	<0.001	Intercept	β_0_	121.07	9.28	13.05	<0.001
				log(ePAL)	β_1_	-39.83	3.84	-10.38	<0.001
	Saliva	0.661	<0.001	Intercept	β_0_	108.52	9.93	10.93	<0.001
				log(ePAL)	β_1_	-33.07	3.92	-8.44	<0.001
Model 2: Age_o_ = β_0 _+ β_1_ log(ePAL) + β_2_ log(ePAL)^2^ n = 37 individuals	Blood	0.753	<0.001	Intercept	β_0_	217.28	74.63	2.91	0.006
				log(ePAL)	β_1_	-122.69	63.89	-1.92	0.063
				log(ePAL)^2^	β_2_	17.53	13.49	1.30	0.203
	Saliva	0.668	<0.001	Intercept	β_0_	208.38	76.32	2.73	0.010
				log(ePAL)	β_1_	-117.87	64.39	-1.83	0.076
				log(ePAL)^2^	β_2_	17.59	13.33	1.32	0.196

The table shows the squared coefficient of correlation (*r*^2^) and statistical significance (*p*) for each model, and the coefficient, standard error, *t-*statistic and statistical significance (*p*) associated with each parameter in the model. The number of individuals used in each analysis is indicated (*n*).

### The behavior of the (CTG)_n_ repeat expansion shows subtle differences among saliva and blood cells in DM1 patients

**Fig 3:**
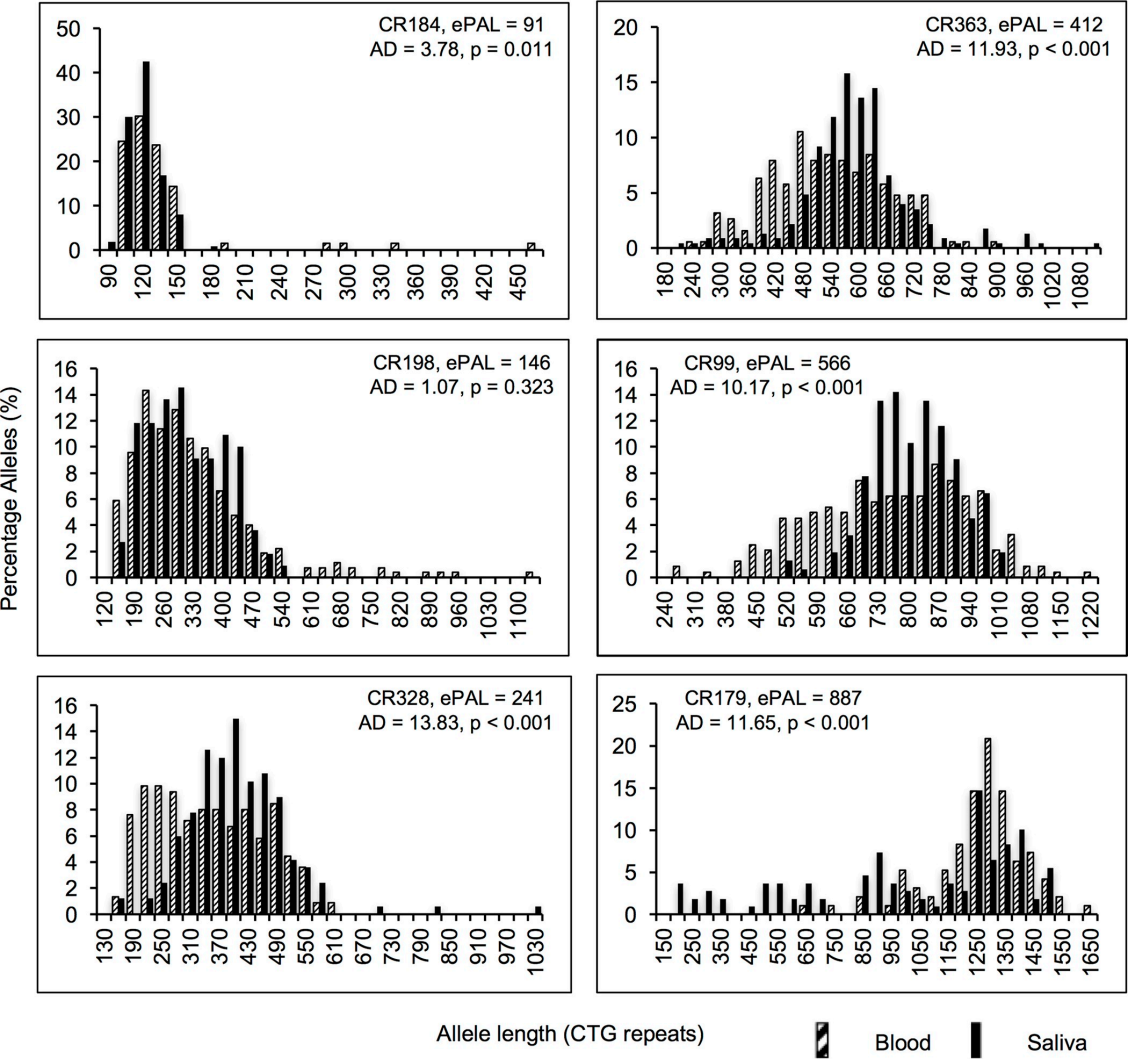
Representative histograms showing the allele distributions in two tissue sources of six DM1 patients. The header indicates the progenitor allele length estimated in blood (ePAL) for each patient and the probability value (*p*) of equal distributions in both sample sources, calculated using the *t*-statistic of Anderson-Darling (AD). Patients with blood ePAL < 150 CTG repeats show similar allele distributions, while non-congenital DM1 patients with an ePAL > 150 CTG repeats showed a higher degree of instability in blood. Congenital cases showed higher levels of instability in saliva than in blood (bottom right).

In order to perform a more detailed quantitative analysis of SI in blood and saliva DNA, we carried out single molecule SP-PCR in 38 DM1 patients. We sized a total of 12,488 mutant alleles with an average of 164 (± 67) molecules per sample (data in S2 Table). The degree of SI (defined as the difference between the 10^th^ and 90^th^ percentile of the total allele distribution) was calculated for each sample. As with the ePAL, the degree of SI measured from both DNA sources was highly correlated (*r* = 0.667, *n* = 38, *p* < 0.001, Fig 2C.). Interestingly, excluding the two congenital cases (CDM) in our study, which showed a clearly different SI pattern (Fig 3), we observed a higher degree of SI in peripheral blood than in saliva (mean SI in blood = 329 repeats; saliva = 250 repeats; *t* = 5.39, df = 35, *p* < 0.001, Fig 2C and 2D and data in S1C Fig).

By investigating the total allele distributions in DM1 patients with small CTG expansions (< 150 CTG repeats in blood ePAL), we observed that in most of the DM1 patients, both cell sources showed similar allele distributions with a positive asymmetry (Fig 3). However, in non-congenital patients with larger alleles (> 150 CTG repeats in blood ePAL), the mutant allele distributions tended to be more symmetrical, being wider for peripheral blood than for saliva cells and, therefore, with the latter distribution immersed within the former (Fig 3). Differences in the boundaries of the total allele distributions were compared (taking the 10^th^ percentile as the lower boundary and the 90^th^ percentile as the upper boundary), and we found that allele distributions in blood and saliva differed to a greater extent in their lower end than in the upper end (mean size difference between the lower boundary = 104.4; upper boundary = 41.9; *t* = 2.67, df = 37, *p* = 0.011, data in S2A Fig).

In order to analyze and compare the major modifiers of SI in the two DNA sources under study, we ran a multivariate regression model that has been previously used for this purpose [16, 18]. As the ePAL measured in blood was considered as the best estimative of the actual PAL, we therefore, used it in the saliva and blood SI models (Table 2). As expected, more than 85% of the SI variation in blood DNA from DM1 patients was explained by a complex synergistic relationship between the ePAL and age at sampling, whereas for DNA obtained from saliva, the same model explained about 72% of the variation in SI (Table 2; data in S2B Fig), suggesting that other unidentified tissue-specific factors, such as relative DNA repair gene expression levels, might be acting as modifiers of the behavior of the CTG repeats in buccal cells.

**Table 2:** Regression models of the relationship between somatic instability (SI) in DNA observed in blood and saliva and the estimated progenitor allele length in blood (ePAL) and the age at sampling (Age_s_).

Model	Source	Adjusted *r*^2^	*p*	Parameter		Coefficient	Standard error	*t*-statistic	*p*
Model 3: log(SI) = β_0 _+ β_1_ log(ePAL) + β_2_(Age_s_) + β_3_ log(ePAL) * (Age_s_) + β_4 _log(ePAL)^2^ + β_5_(Age_s_)^2^ *n* = 38 individuals	Blood	0.859	<0.001	Intercept	β_0_	-28.054	6.599	-4.251	<0.001
				log(ePAL)	β_1_	21.444	4.404	4.869	<0.001
				Age_s_	β_2_	0.179	0.077	2.314	0.027
				log(ePAL) * Age_s_	β_3_	-0.049	0.024	-2.012	0.053
				log(ePAL)^2^	β_4_	-3.825	0.744	-5.139	<0.001
				Age_s_^2^	β_5_	-0.001	0.000	-2.543	0.016
	Saliva	0.717	<0.001	Intercept	β_0_	-27.687	8.743	-3.167	0.003
				log(ePAL)	β_1_	20.471	5.834	3.509	0.001
				Age_s_	β_2_	0.224	0.102	2.196	0.035
				log(ePAL) * Age_s_	β_3_	-0.068	0.032	-2.120	0.042
				log(ePAL)^2^	β_4_	-3.493	0.986	-3.542	0.001
				Age_s_	β_5_	-0.001	0.000	-2.080	0.046

The table shows the squared coefficient of correlation (*r*^2^) and statistical significance (*p*) for each tissue, and the coefficient, standard error, *t*-statistic and statistical significance (*p*) associated with each parameter in the model. The number of individuals used in the analysis is indicated (*n*).

### Neither variant repeats nor methylation levels act as modifiers of SI in the tissues analysed

We next determined the presence or absence of variant repeats (CGG and CCG) within the DM1 (CTG)_n_ repeat and analysed the methylation levels of two CTCF-binging sites flanking the CTG repeat, in order to determine if *cis*-acting modifiers might account for the subtle differences found in the behavior of the CTG repeats between the two tissues [27–29, 33]. The relationship between methylation and SI in DM1 is not yet clear and the presence of variant repeats have been associated with a stabilization of the CTG repeat, which might help to explain the differences we found. However, no CGG or CCG variant repeats were detected in the DNA from blood or saliva in the 38 DM1 patients analysed in this study. This does not exclude the possibility of other rarer variant repeats in these samples. Regarding the methylation study, we considered the DNA samples to be methylated only if the mean methylation of all of the CpGs analysed were ≥ 10%, as measured methylation levels below 10% are considered unreliable [30]. We only detected moderate methylation levels (between 10 to 50%) upstream of the CTG repeat (CTCF1 site) in one of the two CDM cases (being higher in blood than in saliva). Similarly, moderate levels of methylation downstream of the CTG repeat (CTCF2) were also only detected in the two CDM cases analysed in this project and only in blood DNA (Table 3). All the remaining patients showed mean methylation levels in the two analysed CTCF-binding sites lower than 10% in both tissue sources.

**Table 3:** Mean methylation percentage in blood and saliva of congenital cases within two CTCF binding sites.

Site	Sample	Mean methylation (%)	
		Blood	Saliva
CTCF1	CR179	35.92	12.24
	CR189	5.47	5.77
CTCF2	CR179	14.90	2.48
	CR189	12.26	6.28

A total of 11 and 6 CpG sites were analysed for the first (CTCF1) and second (CTCF2) binding sites respectively. Italicized numerals highlight methylated regions. Methylation levels below 10% were considered as baseline levels.

## Discussion

By using Southern blot hybridization of restriction digested genomic DNA from blood, it is possible to measure the modal allele length in blood DNA from DM1 patients. Despite the fact that the allele size thus determined shows a highly significant negative correlation with age of onset, this allele size explains less than 50% of the variation in age of onset [8–10, 12, 34, 35]. We previously demonstrated that these poor correlations are due to the confounding effects of somatic expansion and that by using the ePAL, these clinical correlations could be improved [16, 18]. Notably, the modal allele size measured in skeletal muscle is typically much larger than that observed in blood DNA [19–21]. This observation is consistent with a causal role for somatic expansions driving the tissue specificity of the symptoms. However, repeat lengths in skeletal muscle are usually so large that they cannot be efficiently PCR amplified and need to be measured using Southern blot hybridization of restriction digested genomic DNA. Moreover, modal allele length in muscle provides even poorer age at onset correlations than observed with blood DNA [21]. Again, this can be interpreted as a confounding effect of somatic expansion in driving the modal allele length even further from the PAL in muscle. Thus, other tissues in which the repeat is relatively stable might also be suitable for diagnostic purposes. However, it appears that nearly all other tissues previously assessed in DM1, also contain large somatically acquired expansions [13]. Notably though, the DM1 repeat expansion in cerebellum appears to be even more stable than in blood [29, 36], raising the possibility that estimating the PAL in cerebellum could provide even better genotype to phenotype correlations in DM1. However, cerebellum is not an accessible tissue for performing genetic analyses in DM1 patients. Here, we have revealed that the degree of somatic mosaicism of the expanded CTG repeat in saliva is broadly comparable to that observed in blood DNA and thus represents an excellent source of DNA for genetic studies in DM1. During the initial review of this manuscript, Pesovic et. al. [37] characterized the mutational dynamics of the CTG repeat in blood and buccal cells in a small number of DM1 patients carrying variant repeats in both tissues. They described some features that we also found in our larger cohort: specifically, the progenitor allele length was higher and the levels of somatic instability were lower in buccal cells than in blood, with some differences in the CTG mutational dynamics between both tissues, but with overall much more slower dynamics, triggered by the presence of variant repeats that confers stability to the CTG repeat tract [27, 33]. Obtaining saliva DNA is a much less invasive method than phlebotomy, being of great benefit especially for those patients with fear of needles. This situation could be particularly relevant in children with autism-like symptoms, as commonly encountered in juvenile and congenital DM1 cases [1, 2]. Furthermore, saliva has been widely used for carrying out large population screening studies, a study that could be conducted in DM1 now that we have established the mutational behavior and spectrum of the CTG repeat in saliva, and the justification for which increases as we move toward the delivery of novel therapies.

Previously, it was shown that the lower boundary of the total allele distributions obtained through SP-PCR were conserved over time and between different tissues [15]. In agreement with this, the PALs estimated from the two analysed tissues were highly correlated in our sample set, with very similar lower boundaries in patients with ePAL < 150 CTG repeats. However, we observed that, though correlated, the boundaries were no longer conserved above 150 repeats, where the PAL estimation was consistently higher when analyzing saliva. This suggests that these differences have arisen from tissue-specific mutational dynamics. Interestingly, the ePAL from saliva explained about 66% of the variation in the age of onset, which is slightly lower than the 75% of the variation explained by the ePAL obtained from blood (Table 1). These data suggest that the PAL estimated from blood more accurately reflects the true PAL. Nonetheless, the ePAL measured using saliva DNA still provided much better age at onset correlations than the traditional measurement of the midpoint of the smear obtained through Southern hybridization of blood genomic DNA. Our results thus indicate that saliva could be an appropriate surrogate for performing genetic analyses in DM1. Similar to Pesovic et. al. [37], we also used the 10^th^ percentile of the total allele distribution as an estimation of the PAL as an alternative way for measuring this allele size (data not shown). Although results were similar between ePAL and the 10th percentile (as an estimation of the PAL) in both tissues, measuring the 10th percentile of the total allele distribution is more technically challenging, more time-consuming and more expensive than measuring the ePAL only.

**Fig 4:**
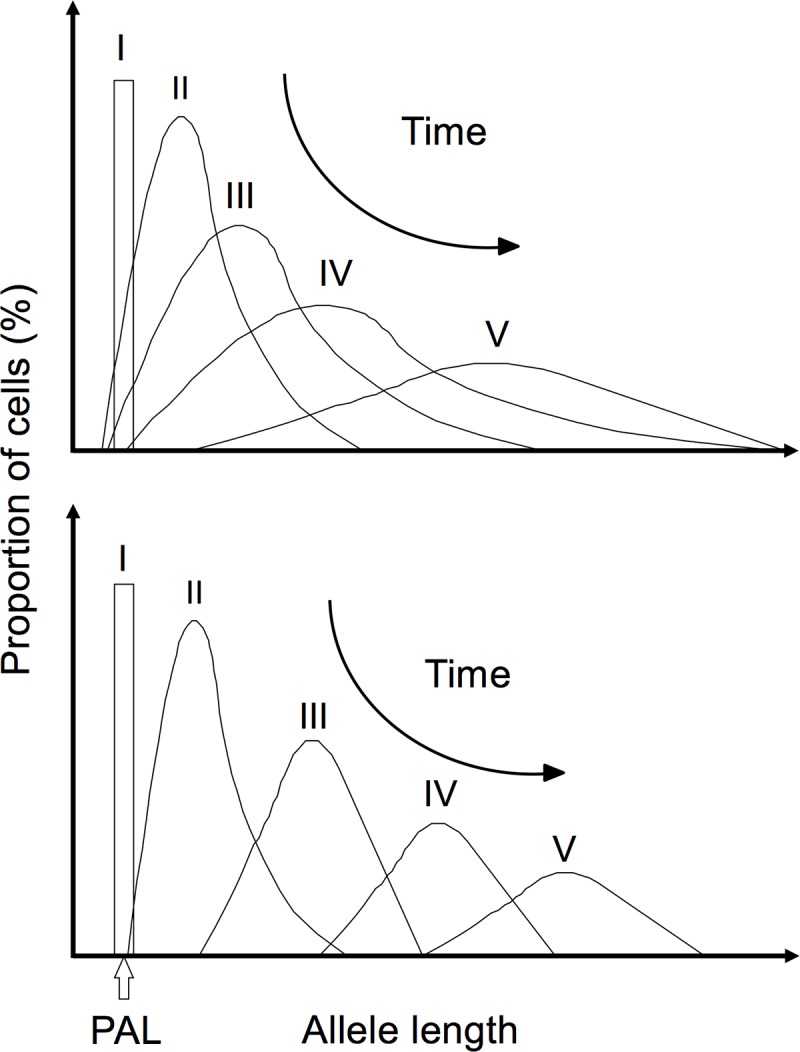
Expansion models of the CTG repeat expansion in blood and saliva. The top model was proposed previously for the mutational dynamics of the CTG repeat expansion in blood DNA (modified from [15]). Data from this study suggests that in saliva (lower model), the rate of expansion/contractions is different than in blood, triggering a faster movement of the lower boundary, a more compact allele distribution and faster progression to a normal distribution than in blood and with larger modal allele length in saliva with time. In both models, as the number of CTGs increases, the mean and modal alleles increase and their frequency decreases with time.

Since it has been previously suggested that CTG•CAG somatic instability starts after the first three months of embryonic development [38], right after the separation of the germ layers that give rise to the tissues represented in the sample sources under study (*i*.*e*., ectoderm for buccal epithelium and mesoderm for hematopoietic cells) [39, 40], it is unlikely that the differences found in the lower boundaries of allele distributions have been caused by an early establishment of embryonic layers with different sizes of mutated alleles. Most likely, this phenomenon could be attributed to parameters in the post-natal mutational dynamics of differentiated tissues. Interestingly, although saliva showed a higher lower boundary and a higher modal allele length, PBLs showed higher levels of SI, providing evidence that the mutational dynamics in different tissues don't just reflect differences in the absolute rate of expansion. Previously using a mathematical modelling approach we revealed that the broad repeat length distributions observed in blood DNA are likely driven by a high frequency of small expansions and a similarly high frequency of small contractions [41]. It is feasible that in buccal cells there is a lower rate of contractions relative to expansions. This would cause a greater upward drift of the lower boundary, but would also result in a narrower range of variants (Fig 4). These observations might be comparable to observations in Huntington disease (HD) expanded CAG repeat mouse models, where a wider population of unstable repeats are observed in striatum in comparison to liver, despite a greater increase in mean allele length in liver [42, 43]. Indeed, a previous study found similar results when comparing the DM1 mutation in blood cells and the HD mutation in buccal epithelium [44]. In this study the estimated mutational rates, including both expansions and contractions, were significantly lower in HD buccal cells than in DM1 blood cells, with a lower occurrence of contractions in the former tissue. Although in this case it is possible that the differences in mutational rates could be attributed to the different genomic context of the implicated unstable repeats, the authors hypothesized that the most suitable explanation could be related to cell type rather than disease type.

The subtle differences observed in the mutational dynamics among tissues might be accounted for by the effect of different *cis-* or *trans-*tissue-specific genetic factors. It is known, that methylation of CTCF binding sites has been previously associated with increased levels of instability of the CAG•CTG repeat associated with spinocerebellar ataxia type 7 (SCA7) [45], and in DM1 methylation seems to vary among tissues, both in humans and transgenic mice [29]. On the other hand, in some unstable repeat diseases such as SCA1, SCA8 and DM1, the purity of the respective causal allele has been associated with SI, while variants within the repetitive tract confer stability to the alleles [27, 33, 46]. In our study, although a higher degree of SI was observed in blood DNA in comparison to saliva, we observed: 1) that the methylation levels of the two (CTG)_n_ repeat flanking CTCF binding sites were conserved among the two sample sources analysed; and, 2) an absence of variant repeats in both of the tissues analysed. This indicates that these factors likely do not contribute to the subtle differences we observed in the somatic mutational dynamics among the tissues analysed.

It should be noted however that the only samples with moderate methylation levels in this study were the two CDM cases analysed, consistent with previous findings that found that this DM1 clinical form preferentially showed methylation flanking the CTG repeat expansion [28, 29, 47, 48], and it has been suggested that methylation could be used as a biomarker for CDM ([47], Morales et al, in preparation). The study carried out by Barbe et. al. [47] and this study, are the only ones that have quantified the levels of methylation flanking the CTG repeat expansion. The difference in the levels of methylation found in both studies could be due to inherent aspects of the used assay. Despite this, and in agreement with what the Barbe et. al. [47] found, we also found increased methylation in CDM cases and upstream of the repeat, with one patient showing higher levels of methylation than the other.

Interestingly, the two CDM cases showed a clearly different SI pattern from that observed in non-CDM cases, bearing a higher proportion of alleles that have acquired very large contractions in saliva than in PBLs. Previous studies in HD mouse models have provided similar observations, showing that mice inheriting large mutated alleles (>500 CAG•CTG repeats) can have a reversion of the expansion/contraction balance in some tissues, with the accumulation of contractions playing an important role in the levels of somatic variation [42]. It remains to be determined whether the apparent increase in large contractions in congenital patients could be attributed to methylation in adjacent CTCF binding sites. A more detailed study of congenital cases could be pertinent, considering the potential therapeutic benefit of inducing contractions with methylating agents [49].

## Conclusions

By comparing two tissue sources, our study has assessed the suitability of employing buccal cells as an alternative tissue source of genetic material to carry out informative molecular analyses in DM1, providing more accurate prognostic information, something that cannot be done with other DM1 tissues due to the excessively large repeat size compared to blood and buccal cells from saliva. Also, the data we present here provide new insights into the CTG tissue-specific mutational dynamics, a feature that is becoming increasingly important in terms of disease severity and progression, and as a target and marker for therapeutic intervention [16, 18, 27, 33, 42, 50, 51]. To achieve effective somatic therapy of the DM1 repeat expansion, careful serial monitoring of therapeutic efficacy and detailed knowledge of the longitudinal CTG mutational dynamics are essential. Clearly, non-invasive access to a readily accessible tissue in which somatic mutational dynamics have been characterized will facilitate inclusion of a large representative DM1 population with the least possible risk.

Although previous studies have already suggested the use of buccal cells for diagnostic purposes in DM1 [52, 53], a detailed quantitative validation through single molecule SP-PCR in order to evaluate the suitability of using saliva instead of blood, which is the standard source for DNA testing in DM1, has not yet been performed in DM1 patients. Even though we found subtle differences in the mutational dynamics in saliva and blood DNA, we provide evidence that the PAL estimation through the SP-PCR assay using DNA obtained from saliva constitutes a good surrogate tissue and less invasive approach for DM1 diagnosis. Our results are particularly relevant given that in some of the main tissues affected in DM1 (such as skeletal muscle), determination of reliable estimates of the PAL is challenging due to the high levels of somatic mosaicism, which potentially compromises the quality of clinical correlations obtained. On the other hand, tissues that have been proven to be especially stable (such as cerebellum) are not accessible, which limits their usefulness for performing routine molecular analysis. As demonstrated here, the use of saliva DNA for these purposes, in combination with SP-PCR, constitutes a useful alternative when the collection of blood samples is not feasible or problematic.

## Supporting information

S1 Table List of oligonucleotides used in this study. List of primers used in this study. BTN refers to a biotin on the 5' end.
(DOCX)

S2 Table Molecular and clinical data. Table contains all molecular data and some clinical information for each sample used in this study.
(XLSX)

S1 Fig **Box plots for the comparison of the modal allele length (A), the progenitor allele length (ePAL) (B) and the degree of somatic instability (SI) (C) from two different DNA sources of the same DM1 patient.** For each measurement, the interquartile ranges (IQR) are indicated as boxes; the medians and means are represented by solid and dotted lines respectively subdividing the boxes; bars indicating the 90^th^ and 10^th^ percentiles are shown as whiskers above and below the box; data points beyond the whiskers are outlying points.
(PDF)

S2 Fig **A. Differences in the boundaries from allele distributions from blood and saliva DNA from 38 DM1 patients.** The 10^th^ percentile of total allele distribution is taken as the lower boundary, whereas the 90^th^ percentile is taken as the upper boundary. The box plot shows a much larger variation on the lower boundary than in the upper boundary between the two tissues. The interquartile ranges (IQR) are indicated as boxes; the medians and means are represented by solid and dotted lines respectively subdividing the boxes; error bars indicating the 90th and 10th percentiles are shown as whiskers above and below the box; data points beyond the whiskers are outlying points. **B. Polynomial relation of the degree of SI in blood and saliva with the age at sampling and the logarithm of the progenitor allele length (ePAL) estimated in blood.** The degree of SI was measured as the difference between the 10^th^ and 90^th^ percentiles of the allele distributions in each sample source. The predicted functions for the polynomial multiple regressions are shown as a mesh.
(PDF)
