# Supplementary material for: Analysis of mutational dynamics at the DMPK (CTG)n locus identifies saliva as a suitable DNA sample source for genetic analysis in myotonic dystrophy type 1
Source: PLoS One. 2019 May 2;14(5):e0216407. doi: 10.1371/journal.pone.0216407 (PMC6497304; doi:10.1371/journal.pone.0216407)
Supplement: S1 Table — List of primers used in this study. BTN refers to a biotin on the 5' end. (DOCX) [file pone.0216407.s001.docx]

| **Region** | **Oligonucleotide** | **Sequence** | **Size (bases)** | **Tm** |
| --- | --- | --- | --- | --- |
|  | PS-Bio-UNIV2 | 5´-[Btn]GGGACACCGCTGATCGTTTA-3´ | 20 | 62.3ºC |
| CTCF-I | PS-DMPK-F3 | 5´-GTTTAGTTTTAGTTTTGTGATT-3´ | 22 | 53.7ºC |
|  | PS-U2-DMPK-R3 | 5´-GACGGGACACCGCTGATCGTTTATCCCR ACTACAAAAACCCTT-3´ | 43 | 59.1ºC |
|  | PS-DMPK-S3 | 5´-GTTTTAGTTTTGTGATT-3´ | 17 | 48.7ºC |
| CTCF-II | PS-DMPK-F4 | 5´-ATTGTAGGTTTGGGAAGGTAGTA-3´ | 23 | 59.8ºC |
|  | PS-U2-DMPK-R4 | 5´-GACGGGACACCGCTGATCGTTTAaattt aacaaaaacaaatttcc-3´ | 45 | 52.9ºC |
|  | PS-DMPK-S4 | 5´-GGTTTGGGAAGGTAGTAA-3´ | 18 | 56.4ºC |
