# Supplementary figures and images for: Analysis of mutational dynamics at the DMPK (CTG)n locus identifies saliva as a suitable DNA sample source for genetic analysis in myotonic dystrophy type 1
Source: PLoS One. 2019 May 2;14(5):e0216407. doi: 10.1371/journal.pone.0216407 (PMC6497304; doi:10.1371/journal.pone.0216407)

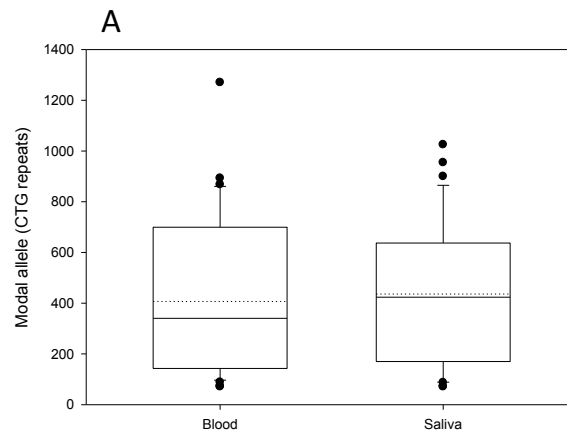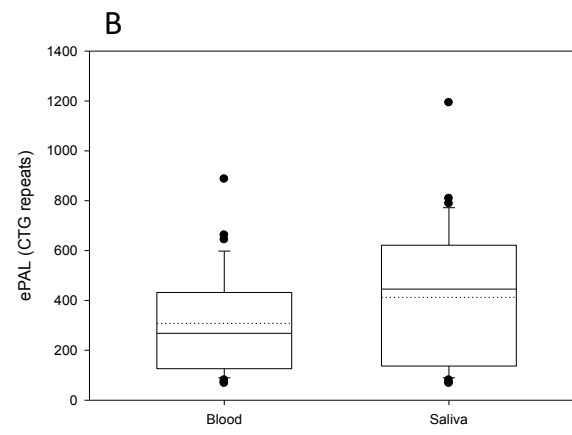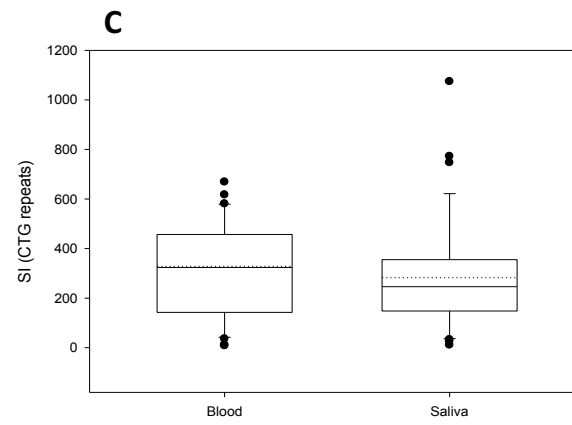

Supplement: S1 Fig — Box plots for the comparison of the modal allele length (A), the progenitor allele length (ePAL) (B) and the degree of somatic instability (SI) (C) from two different DNA sources of the same DM1 patient. For each measurement, the interquartile ranges (IQR) are indicated as boxes; the medians and means are represented by solid and dotted lines respectively subdividing the boxes; bars indicating the 90th and 10th percentiles are shown as whiskers above and below the box; data points beyond the whiskers are outlying points. (PDF) [file pone.0216407.s003.pdf]

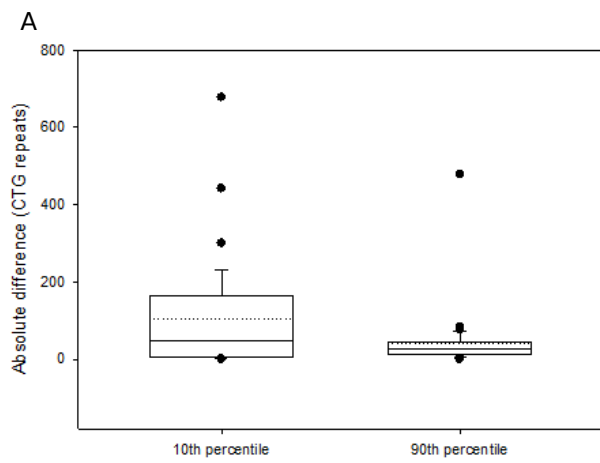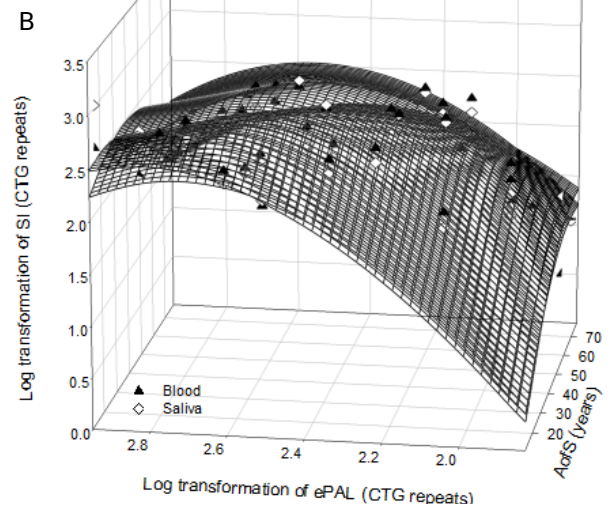

Supplement: S2 Fig — A. Differences in the boundaries from allele distributions from blood and saliva DNA from 38 DM1 patients. The 10th percentile of total allele distribution is taken as the lower boundary, whereas the 90th percentile is taken as the upper boundary. The box plot shows a much larger variation on the lower boundary than in the upper boundary between the two tissues. The interquartile ranges (IQR) are indicated as boxes; the medians and means are represented by solid and dotted lines respectively subdividing the boxes; error bars indicating the 90th and 10th percentiles are shown as whiskers above and below the box; data points beyond the whiskers are outlying points. B. Polynomial relation of the degree of SI in blood and saliva with the age at sampling and the logarithm of the progenitor allele length (ePAL) estimated in blood. The degree of SI was measured as the difference between the 10th and 90th percentiles of the allele distributions in each sample source. The predicted functions for the polynomial multiple regressions are shown as a mesh. (PDF) [file pone.0216407.s004.pdf]
